# Kinetics of SARS-CoV-2 positivity of infected and recovered patients from a single center

**DOI:** 10.1038/s41598-020-75629-x

**Published:** 2020-10-29

**Authors:** Jia Huang, Le Zheng, Zhen Li, Shiying Hao, Fangfan Ye, Jun Chen, Hayley A. Gans, Xiaoming Yao, Jiayu Liao, Song Wang, Manfei Zeng, Liping Qiu, Chunyang Li, John C. Whitin, Lu Tian, Henry Chubb, Kuo-Yuan Hwa, Scott R. Ceresnak, Wei Zhang, Ying Lu, Yvonne A. Maldonado, Doff B. McElhinney, Karl G. Sylvester, Harvey J. Cohen, Lei Liu, Xuefeng B. Ling

**Affiliations:** 1 National Clinical Research Center for Infectious Disease, The Second Affiliated Hospital of Southern University of Science and Technology, Shenzhen, Guangdong Province China; 2 Department of Surgery, Stanford University School of Medicine, Stanford, CA 94305 USA; 3 Department of Cardiothoracic Surgery, Stanford University School of Medicine, Stanford, CA USA; 4 Clinical and Translational Research Program, Betty Irene Moore Children’s Heart Center, Lucile Packard Children’s Hospital, Palo Alto, CA USA; 5 Department of Pediatrics, Stanford University School of Medicine, Stanford, CA USA; 6 Translational Medicine Laboratory, West China Hospital, Sichuan University, Chengdu, China; 7 Department of Bioengineering, University of California at Riverside, Riverside, CA USA; 8 Biomedical Big Data Center, West China Hospital, Sichuan University, Chengdu, China; 9 Medical Big Data Center, Sichuan University, Chengdu, China; 10 Department of Biomedical Data Science, Stanford University, Stanford, CA USA; 11 Department of Medicine, The University of Hong Kong, Hong Kong, SAR China; 12 Department of Health Research and Policy, Stanford University School of Medicine, Stanford, CA USA

**Keywords:** Medical research, Risk factors

## Abstract

Recurrence of severe acute respiratory syndrome coronavirus 2 (SARS-CoV-2) positive detection in infected but recovered individuals has been reported. Patients who have recovered from coronavirus disease 2019 (COVID-19) could profoundly impact the health care system. We sought to define the kinetics and relevance of PCR-positive recurrence during recovery from acute COVID-19 to better understand risks for prolonged infectivity and reinfection. A series of 414 patients with confirmed SARS-Cov-2 infection, at The Second Affiliated Hospital of Southern University of Science and Technology in Shenzhen, China from January 11 to April 23, 2020. Statistical analyses were performed of the clinical, laboratory, radiologic image, medical treatment, and clinical course of admission/quarantine/readmission data, and a recurrence predictive algorithm was developed. 16.7% recovered patients with PCR positive recurring one to three times, despite being in strict quarantine. Younger patients with mild pulmonary respiratory syndrome had higher risk of PCR positivity recurrence. The recurrence prediction model had an area under the ROC curve of 0.786. This case series provides characteristics of patients with recurrent SARS-CoV-2 positivity. Use of a prediction algorithm may identify patients at high risk of recurrent SARS-CoV-2 positivity and help to establish protocols for health policy.

## Introduction

Given the sudden emergence and rapid community transmission of severe acute respiratory syndrome coronavirus 2 (SARS-CoV-2) being observed worldwide, a strategy of social distancing and shelter in place has been widely adopted in an effort to curb the spread of COVID-19 across space and time^1,2^. The quarantining of patients testing positive for SARS-CoV-2 virus is considered mandatory in order to prevent continued viral spread (contagion). In last 6 months, many of COVID-19 patients have since clinically recovered, but it remains unclear the degree to which patients with COVID-19 clinical symptoms and polymerase chain reaction (PCR) test positivity remain contagious and or at risk for disease relapse.

There is concern based on recent findings showing the recurrence of PCR positive results in 116 infected but recovered individuals^3^ that such individuals may be at risk of viral reactivation or reinfection and thus act as carriers and potentially reinfect others. In response, the World Health Organization (WHO) commented that there is currently “no evidence” demonstrating that people who have recovered from the coronavirus are not at risk of re-infection^4^. Thus, varying quarantine strategies have been implemented during the transition of COVID-19 recovering patients from healthcare to non-healthcare settings in this current pandemic.

However, limited information is available regarding viral shedding kinetics and live virus isolation^5^. Variability in PCR methodology will result in different thresholds of the assay for RNA detection, but in one study the SARS-CoV-2 RNA threshold upon PCR testing needs to be greater than 10^6^ copies per sample^6^. To date, there have been no reports of live virus isolation from patients of PCR retest positivity. Importantly, though, pathological evidence of likely viable SARS-CoV-2 virus within pneumocytes was recently obtained from an individual who died unexpectedly from cardiac arrest after showing clinical recovery and three consecutive negative PCR nasopharyngeal (NP) swabs^7^.

In order to assist with pandemic management, a better understanding of the kinetics of SARS-CoV-2 positivity and associated potential (re)infectivity in recovered individuals is critical. Similarly, to assist in managing individuals, setting quarantine strategies and adjudicating limited healthcare resources, prediction models are needed to better define the risk, timing, and relevance of viral PCR positivity with (re)infectivity. Unanswered questions include the time between NP swab test negative and length of effective quarantine, and how infectious cana clinically recovered COVID-19 patient be with recurred SARS-CoV-2 positivity. Pragmatic models should seek to define the kinetics and relevance of PCR-positive recurrence during recovery from acute COVID-19 to better manage risks for prolonged infectivity and reinfection. Thus, limited resources can be concentrated on the isolation of these potential SARS-CoV-2 carriers, with immediate benefits for the patient, the population and the healthcare system.

This study characterizes a single center cohort of consecutive patients with COVID-19 who were followed after recovery and PCR negativity and shown to have one or more recurrent positive PCR results despite strict quarantine. The primary objective was to describe the kinetics of SARS-CoV-2 PCR in a large cohort of infected but recovered individuals and better understand the relevance of recurrent SARS-CoV-2 positivity. The secondary objective was to develop a prediction algorithm to identify patients at high risk of recurrent SARS-CoV-2 positivity and help understand reactivation and reinfection possibilities to establish protocols for health policy.

## Methods

### Study design and participants

All methods were performed in accordance with relevant guidelines and regulations. This study was approved by the Ethics Committee of the Second Affiliated Hospital of Southern University of Science and Technology. Written informed consent was obtained from the participants. For participants under the age of 18 years, written informed consent were obtained from their parents or legal guardians.

The study cohort included consecutive COVID-19 patients admitted to The Second Affiliated Hospital of Southern University of Science and Technology, Shenzhen, China since January 11, 2020. The last follow up date was April 23, 2020. All discharged COVID-19 patients were subjected to strict quarantine at a designated center for 14 days. SARS-CoV-2 quantitative reverse transcription polymerase chain reaction (qRT-PCR) RNA testing was performed every 3–5 days during both hospitalization and quarantine. Follow-up at home quarantine was mandated for an additional 14 days with weekly SARS-CoV-2 qRT-PCR testing. Upon positive NP swab testing, according to the local health policy, these patients were immediately readmitted back to the hospital (Supplementary Fig. S1). Demographic features, comorbidities, clinical symptoms, vital signs, laboratory findings and treatments during the first hospitalization were collected. Sequentially from admission, NP swab testing was performed every 3 days during hospitalization. Reported treatment information included medicines, intensive care unit (ICU) admissions, and respiratory support and ventilation usage. 25 patients from the study population had been previously reported with clinical characteristics, laboratory testing, radiological reports, therapeutic interventions, and clinical outcomes^8^.

### COVID-19 diagnosis, admission, discharge, and quarantine follow-up

Diagnosis, disease severity, treatment and follow-up criteria for COVID-19 infection were based on the preliminary diagnosis and treatment protocols (6th edition) from the National Health Commission of China^9^. Diagnostic criteria for COVID-19 included epidemiological (demographics and comorbidities) history, typical clinical manifestations (fever, respiratory symptoms) and laboratory diagnosis. Pulmonary respiratory syndrome severity was classified into 4 categories: (1) mild: mild respiratory symptoms, no imaging findings of pneumonia; (2) moderate: fever, respiratory symptoms, imaging findings of pneumonia; (3) severe: shortness of breath, respiratory rate > 30 breaths/min, systemic oxygen saturation < 93% at rest on room air, ratio of the systemic arterial partial pressure of oxygen to the fraction of oxygen in inspired air ≤ 300 mmHg, or > 50% progression of radiologic pulmonary lesions over 24 to 48 h; (4) critical: needing mechanical ventilation, extracorporeal membrane oxygenation, or other organ support therapy in the ICU. Discharge criteria included: being afebrile for at least three days, improvement of respiratory symptoms and radiological abnormities in CT or X-ray, and two consecutive negative NP swab tests sampled > 1 day apart. All patients were discharged under strict monitoring conditions: patients were kept for 14 days at a designated center followed by another 14 days at home, in quarantine. All discharged patients were followed up and tested with repeated NP swab PCR analyses. According to the local policy, patients with a positive NP swab test during quarantine were immediately readmitted back to the hospital.

### SARS-CoV-2 tests

Early morning NP swabs were analyzed every 3 days during the hospitalization, every 3–5 days during mandated quarantine at a designated center, and weekly during quarantine at home. Bronchoalveolar lavage washing was sampled from patients with severe illness or undergoing mechanical ventilation. Total RNA was extracted from the clinical specimens using the QIAamp RNA Viral Kit (Qiagen, Heiden, Germany). A qRT-PCR Test Kit (product code. GZ-D2RM25, Shanghai GeneoDx Biotech Co., Ltd) targeting the ORF1ab and N genes of SARS-CoV-2 was used. A cycle threshold (Ct)value less than 37 was interpreted as positive for SARS-CoV-2 RNA^10^.

SARS-CoV-2 antibody Chemiluminescent microparticle immunoassay (CMIA) kit (Innodx, Xiamen, China; catalog no. Gxzz 20203400198) was used to detect SARS-CoV-2 IgM and IgG in plasma. According to the manufacture’s protocol, total antibody detection was based on double-antigens sandwich methodology and the IgM antibody detection was based on μ-chain capture immunoassay. To develop total antibody and IgM antibody assays, recombinant antigens, including the receptor binding domain (RBD) of the SARS-CoV-2 spike protein, were expressed in a mammalian cell system. A recombinant nucleoprotein of SARS-CoV-2 was expressed in *Escherichia coli* to be used as coating antigen of the indirect immunoassays in the IgG antibody kits.

### Outcome

Patients who had a positive NP swab test during post-discharge follow-up and were readmitted to hospital were defined as 'case'. Patients who did not have positive results of NP swab test after discharge were analyzed as ‘control’ patients.

### Statistical analysis and modelling to predict recurrence of PCR positivity

Features including demographics, comorbidities, symptoms, vital signs, laboratory findings, and treatments were assembled for modelling. Univariable analysis was performed on z-score-normalized features, and logistic regression was used to calculate the odds ratios and *P *values for feature filtering. For multivariate model building, a gradient boosting tree algorithm XGBoost was used for constructing a multivariable prediction model. The binary logistic function was used as the objective function. Parameters such as the learning rate (set to 0.3), the minimum child weight (set to 6.8), the maximum tree depth (set to 3), and the subsample fraction of a single tree (set to 0.6) were all tuned by cross validations to avoid over-fitting. The derived model score ranged from 0 to 100 describing the probability of recurrence after discharge. The recurrence prediction model was evaluated using area under the receiver-operating-characteristic curve (ROC AUC), sensitivity, and specificity from tenfold cross-validation. In the cross validation, all the samples were randomly split into 10 equal-sized subgroups, with ratio of cases and controls remaining consistent in each subgroup. Each time 9 out of the 10 folds were picked to train a model the remaining fold was for testing. The process was repeated 10 times so that every sample had a score. Statistical analyses were performed using R software (version 3.5.1).

## Results

### Baseline characteristics

The study comprised a total of 417 consecutive patients confirmed to admit with COVID-19^9^: typical clinical manifestations (fever, respiratory symptoms) and NP swab PCR positivity. These admitted patients were categorized to have mild (N = 16), moderate (N = 309), severe (N = 73), or critical (N = 19) conditions of pulmonary respiratory syndrome. Death occurred 3 of 417(0.7%) patients during initial hospitalization. Of the remaining 414 patients alive, 69 [16.7% (95% CI 13.0–20.3%), case] patients were with recurrence of NP swab PCR positivity and had ≥ 1 readmission(s). Demographics, clinical data, PCR data, and outcomes from the first hospitalization were summarized (Tables 1, 2). Statistically significant differences between case and control patients were observed for patient age, body mass index, and clinical severity of pulmonary respiratory syndrome during the first hospitalization. Case patients were generally younger than the controls (*P* value < 0.001). The majority (93%) of case patients had mild or moderate pulmonary respiratory syndrome at the first admission, and had respiratory symptoms including cough (33.2%) and increased sputum (25%) at the recurrence of PCR positivity. Ct values measured at the 1st hospitalization were similar in the case and control patients (Supplementary Fig. S2A). 67/69 NP-swab-PCR-positive recurring patients were readmitted with mild disease while 2/69, negative for other non-COVID-19 infections, had typical COVID-19 disease manifestations including fever and respiratory symptoms.

**Table 1:** Demographics and baseline characteristics of COVID-19 patients with (i.e. the case group) and without (i.e. the control group) recurring SARS-CoV-2 positivity.

Characteristics	Control, N = 345	Case, N = 69	*P* value
**Age, n (%)**			< 0.001
0–29 years	47 (13.6)	23 (33)	
30–54 years	164 (47.5)	34 (49)	
55–86 years	134 (38.8)	12 (17)	
Male, n (%)	167 (48.4)	28 (41)	0.2
BMI, kg/m^2^	23.2 (21.3, 25.6)	21.9 (20.0, 24.5)	0.03
**COVID-19 severity at 1st admission, n (%)**			0.008
Mild	13 (3.8)	3 (4)	
Moderate	248 (71.9)	61 (88)	
Severe	68 (19.7)	5 (7)	
Critical	16 (4.6)	0 (0)	
**Clinical history, n (%)**			
Hypertension	71 (20.6)	14 (20)	0.9
Diabetes	31 (9.0)	3 (4)	0.2
Coronary heart disease	25 (7.2)	1 (1)	0.1
Cancer	6 (1.7)	0 (0)	0.6
Chronic lung disease	14 (4.1)	2 (3)	0.9
Chronic liver disease	11 (3.2)	2 (3)	0.9

*COVID-19* Coronavirus disease 2019, *SARS-CoV-2* severe acute respiratory syndrome coronavirus 2.

**Table 2:** Clinical characteristics, laboratory tests, treatments, and outcomes of COVID-19 patients with (i.e. the case group) and without (i.e. the control group) recurring SARS-CoV-2 positivity.

Characteristics	Control, N = 345	Case, N = 69	*P* value
**Symptoms, n (%)**			
Fever	228 (66.1)	43 (62)	0.6^&^
Cough	33 (9.6)	28 (41)	< 0.0001^&^
Sputum	15 (4.3)	15 (22)	< 0.0001^&^
Dizziness	9 (2.6)	3 (4)	0.4^&^
Headache	6 (1.7)	7 (10)	0.002^&^
NP soreness	14 (4.1)	9 (13)	0.007^&^
Shortness of breath	2 (0.6)	1 (1)	0.9^&^
Tightness	9 (2.6)	4 (6)	0.2^&^
Bloating	2 (0.6)	0 (0)	0.9^&^
Diarrhea	8 (2.3)	3 (4)	0.4^&^
Fatigue	26 (7.5)	8 (12)	0.3^&^
Chest pain	2 (0.6)	1 (1)	0.9^&^
Muscle or body aches	49 (14.2)	2 (3)	0.008^&^
Chills	5 (1.4)	5 (7)	0.01^&^
Nausea and vomiting	3 (0.9)	1 (1)	0.5^&^
**Imaging feature, n (%)**			
Lung consolidation	68 (19.7)	11 (16)	0.5^&^
Ground-glass opacity	282 (81.7)	60 (87)	0.4^&^
Pulmonary infiltration	255 (73.9)	58 (84)	0.2^&^
Pleural effusion	13 (3.8)	2 (3)	0.9^&^
**Medication treatment, n (%)**			
Methylprednisolone	91 (26.4)	8 (12)	0.008^&^
Immunoglobulin	97 (28.1)	8 (12)	0.004^&^
Tocilizumab	9 (2.6)	0 (0)	0.4^&^
Oseltamivir	54 (15.7)	7 (10)	0.3^&^
Ribavirin	71 (20.6)	9 (13)	0.2^&^
Interferon	287 (83.2)	54 (78)	0.4^&^
Lopinavir/ritonavir	271 (78.6)	49 (71)	0.2^&^
Arbidol	102 (29.6)	11 (16)	0.03^&^
Favipiravir	32 (9.3)	3 (4)	0.2^&^
Hydroxychloroquine sulfate	21 (6.1)	5 (7)	0.8^&^
Antibiotics	69 (20)	7 (10)	0.06^&^
**Supporting treatment, n (%)**			
High-flow nasal cannula oxygen therapy	16 (4.6)	1 (1)	0.3^&^
Non-invasive ventilation	27 (7.8)	0 (0)	0.01^&^
Invasive ventilation	16 (4.6)	0 (0)	0.09^&^
Extracorporeal membrane oxygenation	2 (0.6)	0 (0)	0.9^&^
Continuous renal replacement treatment	5 (1.4)	0 (0)	0.6^&^
**Blood routine, n (%)**			
Hemoglobin, < 13.7 g/dL (male) < 11.9 g/dL (female)	31 (9.0)	4 (6)	0.5^&^
**Total white blood cell count**			
< 3.5 × 10^9^/L	58 (16.8)	8 (12)	0.4^&^
> 9.5 × 10^9^/L	11 (3.2)	2 (3)	0.9^&^
Lymphocyte count, < 1.1 × 10^9^/L	103 (29.9)	12 (17)	0.04^&^
Neutrophil count, > 6.3 × 10^9^/L	16 (4.6)	2 (3)	0.7^&^
Platelet count, < 125 × 10^9^/L	29 (8.4)	8 (12)	0.4^&^
**Blood biochemistry, n (%)**			
Sodium, < 135 mmol/L	21 (6.1)	4 (6)	0.9^&^
Potassium, < 3.5 mmol/L	29 (8.4)	4 (6)	0.6^&^
Urea, > 9.5 mmol/L	3 (0.9)	1 (1)	0.5^&^
Creatinine, > 111, μmol/L	6 (1.7)	0 (0)	0.6^&^
Albumin, < 40 g/L	51 (14.8)	5 (7)	0.1^&^
ALT, > 45 U/L	27 (7.8)	6 (9)	0.8^&^
AST, > 45 U/L	36 (10.4)	1 (1)	0.01^&^
Lactate dehydrogenase, > 250 U/L	113 (32.8)	12 (17)	0.01^&^
Creatine kinase, > 310 U/L	7 (2.0)	2 (3)	0.6^&^
**Infection-related biomarkers, n (%)**			
Erythrocyte sedimentation rate, > 20 mm/h	151 (43.8)	22 (32)	0.08^&^
Interleukin 6, > 7 p/mL	97 (28.1)	11 (16)	0.04^&^
**Procalcitonin**			0.04^&^
0.1 ng/mL	335 (97.1)	61 (88)	
≥ 0.1 to < 0.25 ng/mL	10 (2.9)	4 (6)	
≥ 0.25 to ≤ 0.5 ng/mL	0 (0)	1 (1)	
> 0.5 ng/mL	0 (0)	1 (1)	
C-reactive protein, > 8 mg/L	151 (43.8)	21 (30)	0.04^&^
**Coagulation function, n (%)**			
Prothrombin time, ≥ 16 s	3 (0.9)	1 (1)	0.5^&^
**D-dimer**			0.5^&^
≤ 0.5 μg/mL	265 (77.0)	55 (80)	
> 0.5 and ≤ 1 μg/mL	58 (16.8)	8 (12)	
> 1 μg/mL	22 (6.4)	4 (6)	
**SARS-CoV-2 antibody at discharge**^a^			0.23^&^
Having IgG/IgM tested, n	114 (33.0)	40 (58)	
IgG positive, n (%)	113 (99.1)	40 (100)	
IgM positive, n (%)	55 (48.2)	30 (75)	
**Clinical outcomes**			
ICU admission	34 (9.9)	0 (0)	0.002^&^
Length of hospitalization, (day)	20 (12)	20 (12)	0.5^#^

Data are presented in form of n (%) or median (IQR), unless otherwise stated. For statistical analyses, the Mann–Whitney U test was used to compare continuous variables and Fisher’s exact test was performed to compare categorical variables between groups.

*COVID-19* Coronavirus disease 2019, *SARS-CoV-2* severe acute respiratory syndrome coronavirus 2.

^#^*P* value was calculated by the Mann–Whitney U test.

^&^*P* value was calculated by the Fisher exact test.

^a^IgG/IgM tests were performed at discharge since February 12, 2020.

### Patient clinical history

**Figure 1:**
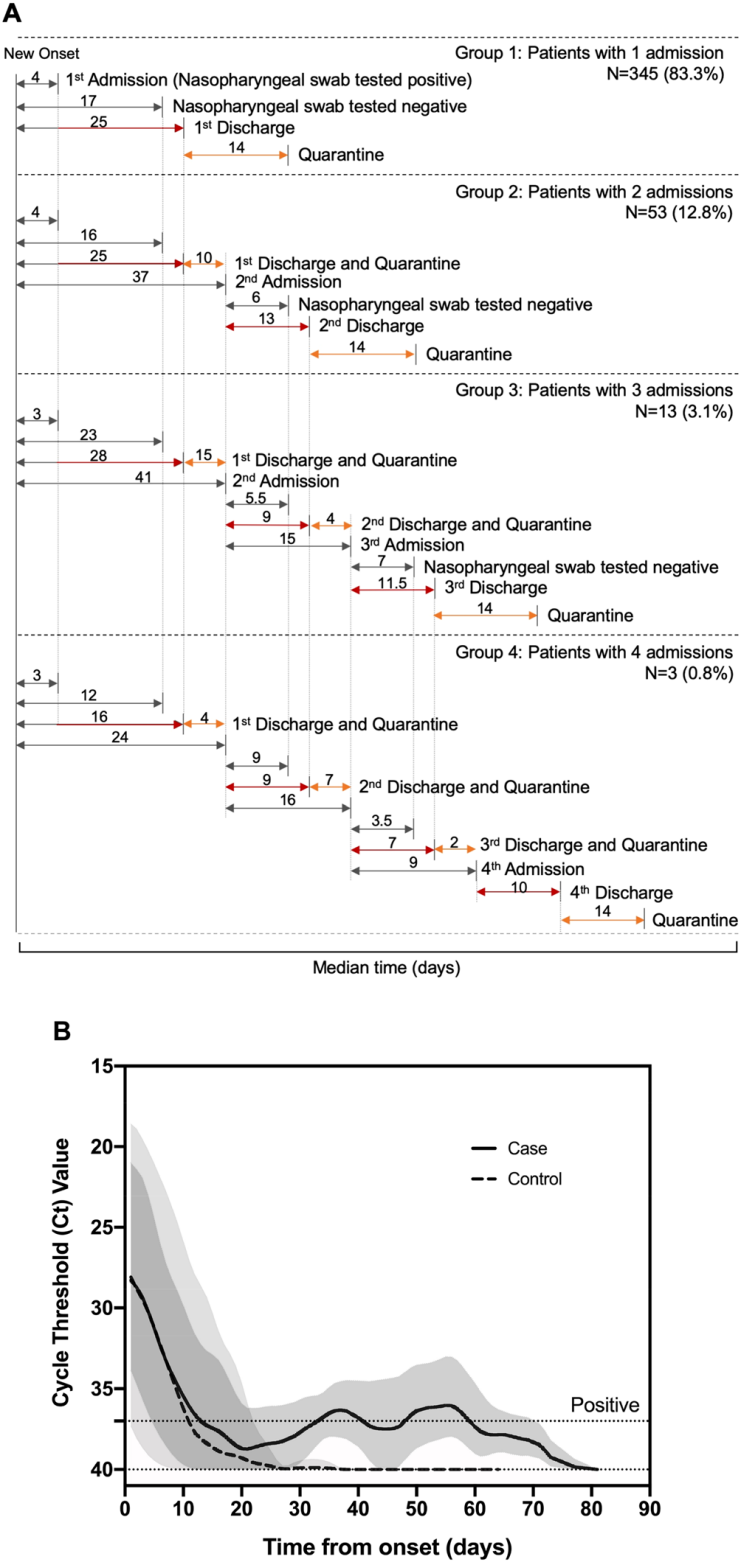
Kinetics of SARS-CoV-2 positivity in infected but recovered COVID-19 patients. (**A**) The COVID-19 timeline summarizes the median duration (days) from the onset of symptoms to clinical events and recurrence of NP swab PCR positivity. Patients are grouped by the number of (re)admissions. Clinical events include admission, NP swab tested negative, discharge, and quarantine ended due to either retest positive or release to home. (**B**) Trends of SARS-CoV-2 PCR positivity over time. The case and control lines show the trends of the variation over time in diagnostic tests for detection of SARS-CoV-2 infection, using smoothing splines. *COVID-19* Coronavirus disease 2019, *SARS-CoV-2* severe acute respiratory syndrome coronavirus 2, *PCR *polymerase chain reaction, *NP* nasopharyngeal.

The timeline of clinical events including admission, discharge, quarantine and readmission are summarized (Fig. 1A, cases are divided into subgroups according to number of readmissions; Supplementary Figs. S2B and S3).A total of 69 patients, with one (N = 53), two (N = 13), or three (N = 3) recurring positive NP swab tests, had mild/moderate disease during the first admission (Fig. 1A).As of April 23rd, 67 of the 69 total cases have been considered clinically free of COVID-19, while 2 patients still remained within their second hospitalization. The median time (days) from new onset of symptoms to either the first positive or the first negative NP swab PCR test was 3 or 12 days, respectively. There is no significant difference in days between the new onset of symptoms and the first NP swab test negative between the case and control groups (Supplementary Fig. S2B). Distribution of time (days) from new onset of symptoms to the first positive NP swab test was analyzed (Supplementary Fig. S4).

### Trending of the recurrent SARS-CoV-2 positivity

The kinetics of the NP swab PCR positivity, likely representingthe variation over time in diagnostic tests for detection of SARS-CoV-2 infection, is as shown in Fig. 1B. A pattern of decreased time intervals, from SARS-CoV-2 2PCR negative to positive recurrence, was observed: 69 recovered individuals were found to be with first time recurrent PCR positivity after a median of 19 days; 16 recovered ones were found to be with second time recurrent PCR positivity after a median of 8.5 days; and 3 recovered ones were found to be with third time recurrent PCR positivity after a median of 5.5 days.

**Figure 2:**
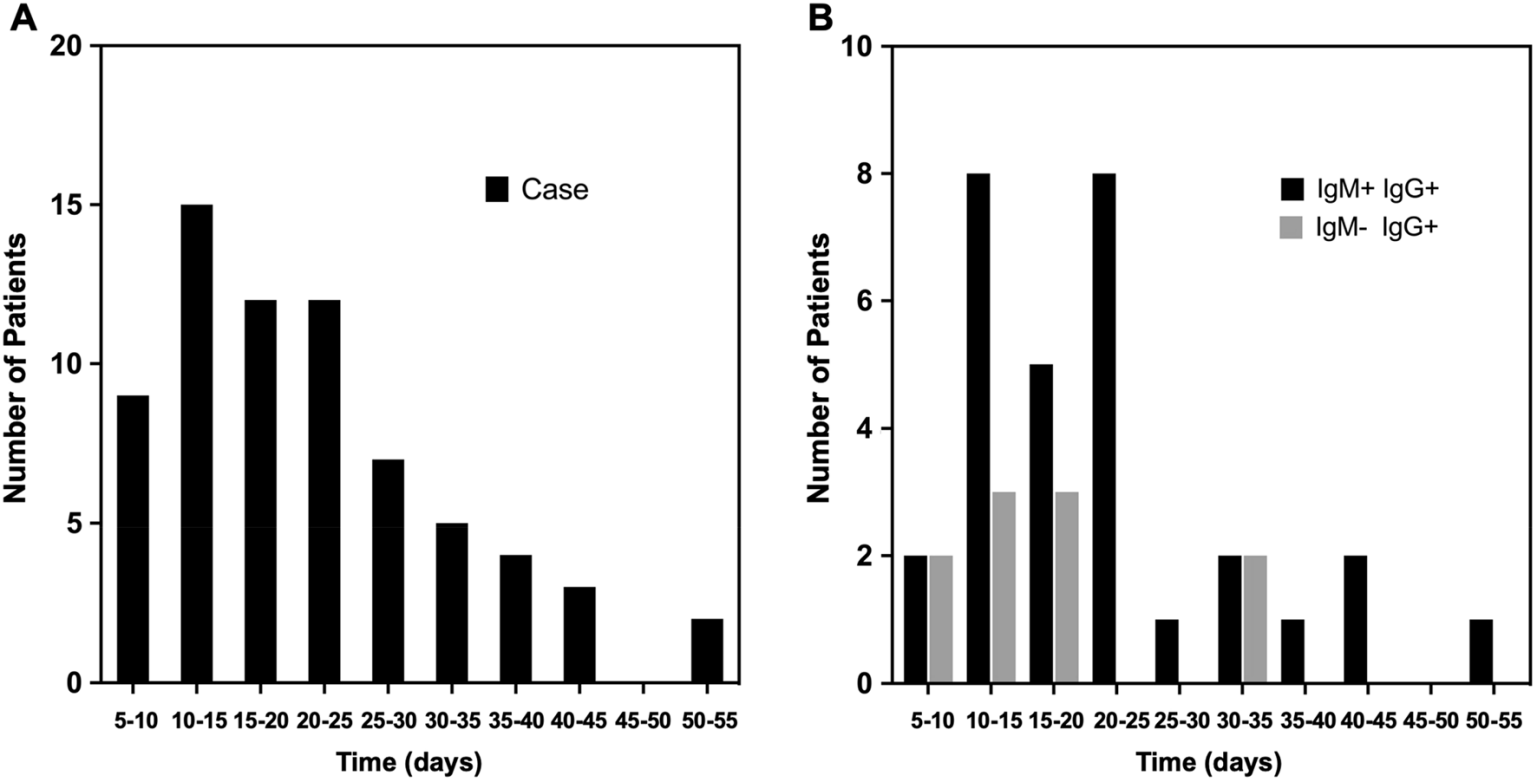
Histogram analyses: distribution of COVID-19 patients according to the duration (days) between the first negative and the first recurring positive NP swab test results. (**A**) COVID-19 patients with recurrence of PCR positivity (the case group). (**B**) The subset (N = 40) of the case group who had IgM and IgG antibody testing performed at the initial discharge. All case patients with the antibody tests were IgG positive. *COVID-19* Coronavirus disease 2019, *PCR *polymerase chain reaction, *NP* nasopharyngeal.

Histogram analysis was used to summarize the distribution of the PCR retest positive patients as a function of time (days) between the first NP swab test negative and test positive results (Fig. 2A, median 19 days, range 6–52 days). Within the case group, 70% retested positive within 5–25 days after the first negative test, with a peak occurring at 10–15 days (22%).

### Serology results in case and control patients

Of note, a subset of 154 patients had IgG/IgM antibody testing at the initial discharge, among which 85 and 153 were IgG and IgM positive, respectively. 1/154 had repeated negative antibody tests (N = 5) of both IgM and IgG against SARS-CoV-2. This suggests thatconvalescent patients, including aging populations^6^, may fail to developSARS-CoV-2 specific IgM and IgG. Of the 154 patients tested, 40 (100%) of the case group were IgG positive, and 30 (75%) were IgM positive (Fig. 2B).

### Predicting recurrence of SARS-CoV-2 positivity

The model was built with 69 cases and 345 controls. Eighteen clinical factors were selected based on *P* values and utilized by the XGBoost algorithm for the final model (Supplementary Table S1 and Supplementary Text S1).

**Figure 3:**
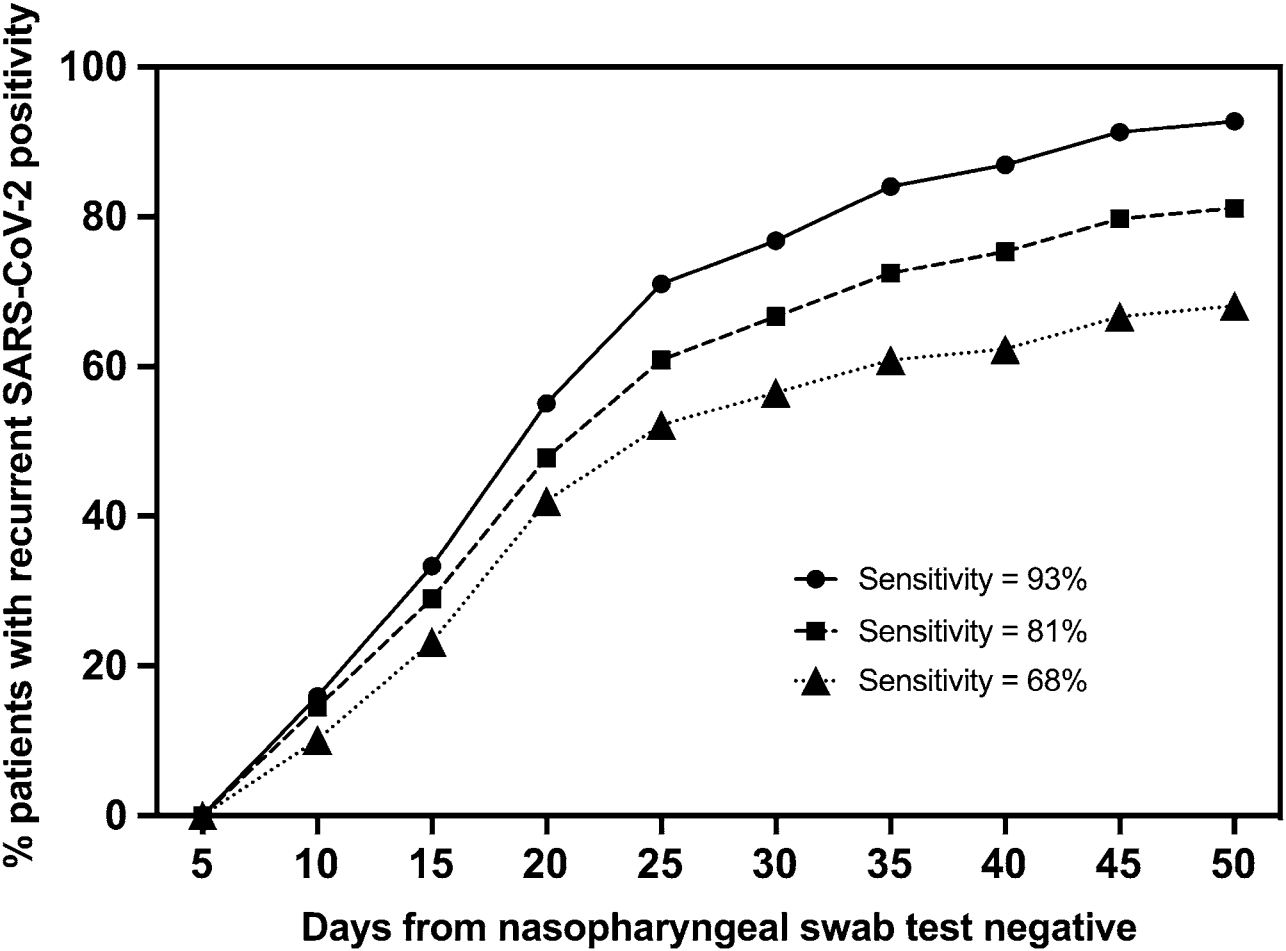
High-risk patients and their recurrent SARS-CoV-2 positivity. The X-axis indicates the duration of time (days) from the first negative NP swab test to the first positive retest during strict post-discharge quarantine. The Y-axis indicates the percentage of high-risk patients who were retest positive within the specified duration of time after the first negative test. Three thresholds for high-risk patients were applied, giving an overall sensitivity of 93%, 81%, and 68%, respectively. *SARS-CoV-2* Severe acute respiratory syndrome coronavirus 2.

The prediction model displayed an overall AUC of 0.786 (95% CI 0.728, 0.843) based on tenfold cross-validation (Supplementary Table S2). To determine the performance and demonstrate the utility of the model, an analysis of ‘days to PCR positivity’ of high-risk patients was under-taken. This analysis supports our hypothesis that our prediction is feasible and may give actionable information at the time of the first of the consecutive NP swab negative tests during the hospitalization (Fig. 3) or at the discharge time (Supplementary Fig. S5).

## Discussion

Given the rapidly evolving COVID-19 pandemic, it is critical to better define the kinetics and relevance of PCR results to understand how long SARS-CoV-2 infected individuals remain infectious and if they are at risk for recurrent disease or reinfection as has been recently suggested^11,12^. Further, it would be helpful to understand which infected individuals would be at highest risk for variance in PCR testing and which may be at higher risk for recurrence or reinfection. To our knowledge, this study is among the first to conduct a comprehensive assessment of PCR kinetics with regular serial viral RNA testing in the setting of strict quarantine. We also propose a case finding model to identify patients at high risk for SARS-CoV-2 viral retest positivity to assist in the establishment of protocols for COVID-19 health policy.

The 16.7% NP swab retest positive rate has not been previously reported. Given the mandated quarantine for discharged patients at a designated center for a minimum of 14 days followed by another 14 days at home (total 28 days of strict social distancing, shelter-in-place), the observed overall rate and multiple recurrence of positive viral testing are unlikely to be from re-infection. This contrasts with the recently case reports in South Korea^11,12^, where in recovered patients were home quarantined and recurrence of PCR positivity there could have arisen from either dormant virus reactivation or re-infection.

COVID-19 patients with multiple recurrences of PCR positivity have not been previously reported. Thirteen and three cases recurred two and three times with PCR positivity, respectively, making it likely that the re-emergence of PCR positivity is due to low levels of viral RNA right at the level of detectability by PCR assay and therefore is at times below this threshold and other times above. The Ct values observed in the samples after the initial positive samples are all high suggesting potential non-viable viral RNA. However, the reimmigration of the virus from the lower to the upper respiratory tract cannot be ruled out^7^. Importantly, it must be noted that the positive SARS-CoV-2 test does not equate with infectivity. Since the current standard for SARS-CoV-2 test positivity is predicated on viral load detection by PCR^6^, sample testing in the case cohort of < 10^6^ copies per sample may not represent a live virus isolate. Unless equipped with a live virus isolate, we cannot be certain whether these retest positive patients were capable of infecting others (contagious) given that they were quarantined at a designated center. To address the possibility of false positive SARS-CoV-2 PCR results, every retest was validated by examinations at both laboratories of Center for Disease Control in Shenzhen and the Second Affiliated Hospital of Southern University of Science and Technology. 95% tests were consistent^13^. The PCR retest positivity cannot differentiate re-infectivity, relapse, and dead-viral RNA detection. Handoff note, a postmortem pathological study found that SARS-CoV-2-viruses still remained in pneumocytes of a recovered COVID-19 patient with a negative PCR test result^7^.

Ninety-three percent of the retest positive patients had mild or moderate severity disease during their first hospitalization. No obvious trending was found in this case series between the initial viral load and first admission symptoms as has been previously reported^6^, e.g. temperature (Supplementary Fig. S6), consistent with previous findings^14^. The two readmitted febrile patients with typical clinical manifestations satisfying the COVID-19 diagnostic and admitting criteria^9^ may have been capable of transmission given the presence of positivity with both viral load testing and COVID-19 admission symptoms.

The key rationale of the local health policy for implementing the described strict center-based quarantine is to prevent transmission. Our findings demonstrate that the effectiveness of this quarantine strategy in the management of the pandemic may be crucial in minimizing late transmission. They also provide insight into the case management of retested positive patients with or without typical COVID-19 symptoms^3,14–28^ and immunity.

Determining the kinetics of NP PCR and those at highest risk for recurrence would provide valuable information to understand if these individuals pose a public health risk for infecting others or individual risk for disease reactivation. Given that the majority of the individuals who were shown to have recurrent NP PCR positive had mild disease, this suggests that the amount of virus is at the threshold of detection and the discordant results may be a result of detectability of the assay.

Determining those at highest and lowest risks of recurrence of PCR positivity may be an essential component of any strategy to enable readmissions, guide interventions, prevent transmissions, and optimize limited care resource utilization. The analysis described herein builds on our previously validated machine-learning models to predict hospital readmission to develop COVID-19 readmission risk prediction tool^29^. The algorithm described here enables different case management strategies upon risk stratification, and facilitates the incorporation of different assumptions about the impact of the intervention and quarantine (Supplementary Fig. S1). At discharge, those patients flagged by the algorithm to be at highest risk may need different follow-up or different screening strategies. Most critically, the time and treatment-dependent trajectory to completely clear dormant and/or live SARS-CoV-2 needs to be established.

The driving factors for this model can be collected in any inpatient settings, and therefore it is portable internationally for immediate validation to provide clinical utility in the current COVID-19 pandemic.

### Limitations

This study has several limitations. First, the study population only included COVID-19 patients within a single center in Southern China during COVID-19 pandemic. The imbalance between case and control numbers may compromise the comparability of both groups, limiting the translation of these real-world clinical observations into evidence. Second, the IgM/IgG tests were not performed on all patients due to the late introduction of the antibody test. Thirdly, the low specificities shown in Supplementary Table S2 revealed that the model would pick up false positives when predicting patients at high risk of positive retesting. Given that the local policy targeted to identify retest positive patients, the high sensitivity values were pursued during the modeling threshold determination, resulting relative low specificity value and “false positives”. We cannot rule out the possibility of “false positives” due to the small single-site cohort size. A further validation with a multi-center trial of powered sample size should ultimately deliver the targeted sensitivity and specificity. Fourthly, and most importantly, the(re)infectious ability of SARS-CoV-2 measured positive by the NP swab test needs to be quantified. Live virus isolation from retest positive patients still requires further effort in order to quantify risk associated with recurrence of PCR positivity at a viral RNA level.

## Conclusion

This case series demonstrates that recurrence of SARS-CoV-2 RNA positivity is relatively common, with a 16.7% rate, in recovered COVID-19 patients. Younger patients with less severe initial illnesses were more likely to retest positive. More information will be required to understand the relevance of these findings, which will be critical for informing the care management of the pandemic. Our prediction algorithm to identify patients at high risk of recurrence of SARS-CoV-2 positivity may establish protocols for better COVID-19 health policy.

## Supplementary information

Supplementary Information.

## Data Availability

Data are available upon request from the corresponding author.
