# Supplementary material for: Kinetics of SARS-CoV-2 positivity of infected and recovered patients from a single center
Source: Sci Rep. 2020 Oct 29;10:18629. doi: 10.1038/s41598-020-75629-x (PMC7596704; doi:10.1038/s41598-020-75629-x)
Supplement: Supplementary file 1 — Supplementary Information. [file 41598_2020_75629_MOESM1_ESM.docx]

**Kinetics of SARS-CoV-2 positivity of infected and recovered patients from a single center**

Jia Huang, MD^1,2#^, Le Zheng, PhD^3,4#^, Zhen Li, PhD^2#^, Shiying Hao, PhD^3,4#^, Fangfan Ye, MD^1,2#^, Jun Chen, MD^1*^, Hayley A. Gans, MD^5^, Xiaoming Yao, MD, PhD^6^, Jiayu Liao, PhD^7^, Song Wang, BS^1^, Manfei Zeng, MD^1^, LipingQiu, MD^1^, Chunyang Li, PhD^8,9^, John C. Whitin, PhD^5^, Lu Tian, PhD^10^, Henry Chubb, MBBS, PhD^5^, Kuo-Yuan Hwa, PhD^11^, Scott R. Ceresnak, MD^5^, Wei Zhang, MD^8,9^, Ying Lu, PhD^10^, Yvonne A. Maldonado, MD^5,12^, Doff B. McElhinney, MD^3,4^, Karl G. Sylvester, MD^2^, Harvey J. Cohen, MD, PhD^5^, Lei Liu, MD^1$^, Xuefeng B. Ling, PhD^2,4$*^

^1^National Clinical Research Center for Infectious Disease, The Second Affiliated Hospital of Southern University of Science and Technology, Shenzhen, Guangdong Province, China

^2^Department of Surgery, Stanford University School of Medicine, Stanford, CA, United States

^3^Department of Cardiothoracic Surgery, Stanford University School of Medicine, Stanford, CA, United States

^4^Clinical and Translational Research Program, Betty Irene Moore Children's Heart Center, Lucile Packard Children’s Hospital, Palo Alto, CA, United States

^5^Department of Pediatrics, Stanford University School of Medicine, Stanford, CA, United States

^6^Translational Medicine Laboratory, West China Hospital, Sichuan University, Chengdu, China

^7^Department of Bioengineering, University of California at Riverside, Riverside, CA, USA

^8^Biomedical Big Data Center, West China Hospital, Sichuan University, Chengdu, China

^9^Medical Big Data Center, Sichuan University, Chengdu, China

^10^Department of Biomedical Data Science, Stanford University, Stanford, CA, United States

^11^Department of Medicine, The University of Hong Kong, Hong Kong SAR, China

^12^Department of Health Research and Policy, Stanford University School of Medicine, Stanford, CA, United States

^#^Jia Huang, Le Zheng, Zhen Li, Shiying Hao, Fangfan Ye, and Jun Chen contributed equally

^$^Lei Liu, and Xuefeng Ling contributed equally

^*^Corresponding Authors:

Xuefeng B. Ling, Stanford University School of Medicine, Stanford, CA 94305, USA; Tel: 650-427-9198; Fax: (650)-723-1154; Email: [bxling@stanford.edu](mailto:bxling@stanford.edu)

Running title: SARS-CoV-2 positivity of patients

**Supplementary material**

**Figure S1**.  Clinical workflow of the COVID-19 patient full cycle management. A schematic chart showing the patient status (location) from the beginning to the end of the study. ​COVID-19: Coronavirus disease 2019.

**Figure S2**. (A)These column charts show the cycle threshold (Ct) value of NP swab qRT-PCR tests of case and control groups: first and second time of SARS-CoV-2 positivity. A Ct value less than 37 was interpreted as positive for SARS-CoV-2 RNA. (B) Percentage of patients remaining NP swab tested positive as days after the new onset of symptoms.SARS-CoV-2: Severe acute respiratory syndrome coronavirus 2.qRT-PCR : Quantitative reverse transcription polymerase chain reaction. NP: Nasopharyngeal.

**Figure S3**. The timeline depicts the median number of days from the onset of symptoms to clinical events. Patients are separated into two groups, with (i.e. the case group) and without (i.e. the control group) recurrence of NP PCR positivity. Clinical events include admission, NP swab tested negative, discharge, and quarantine ended due to either retest positive or release to home. ​NP: Nasopharyngeal. PCR: Polymerase chain reaction.

**Figure S4**. These column charts demonstrate the distribution of case and control group individuals as a function of the number of days from the onset of symptoms to the first positive NP swab results. ​NP: Nasopharyngeal.

**Figure S5**. High-risk patients had NP swab positive retests. The X-axis represented the duration of time (days) from the initial discharge date to the first positive retest during strict post-discharge quarantine. The Y-axis shows the percentage of high-risk patients who were retested as positive and readmitted to the hospital within the specified duration after discharge. Three thresholds for high-risk patients were applied, giving an overall sensitivity of 93%, 81%, and 68%, respectively. ​​NP: Nasopharyngeal.

**Figure S6**. This scatterplot demonstrates the relationship between SARS-CoV-2 RNA cycle threshold (Ct) value and body temperature at admission for patients, defined by NP swab PCR detection, with [i.e. the case group; solid circles] and without (i.e. the control group; open diamonds) recurrence of PCR positivity. The vertical dotted line represents the threshold of high body temperature (> 37.2°C). ​SARS-CoV-2: Severe acute respiratory syndrome coronavirus 2. ​​NP: Nasopharyngeal.PCR: Polymerase chain reaction.

**Table S1**. Model performance in predicting future recurrence of SARS-CoV-2 positivity. A) The key factors predicting subsequent recurrence of PCR positivity. Ct: Cycle threshold. CI: confidence interval. ​SARS-CoV-2: Severe acute respiratory syndrome coronavirus 2. PCR: Polymerase chain reaction.

**Table S2.** Model performance: Sensitivity and specificity at different thresholds.

**Text S1.** Features predicting future recurrence of SARS-CoV-2 positivity. SARS-CoV-2: Severe acute respiratory syndrome coronavirus 2.

**FigureS1**.


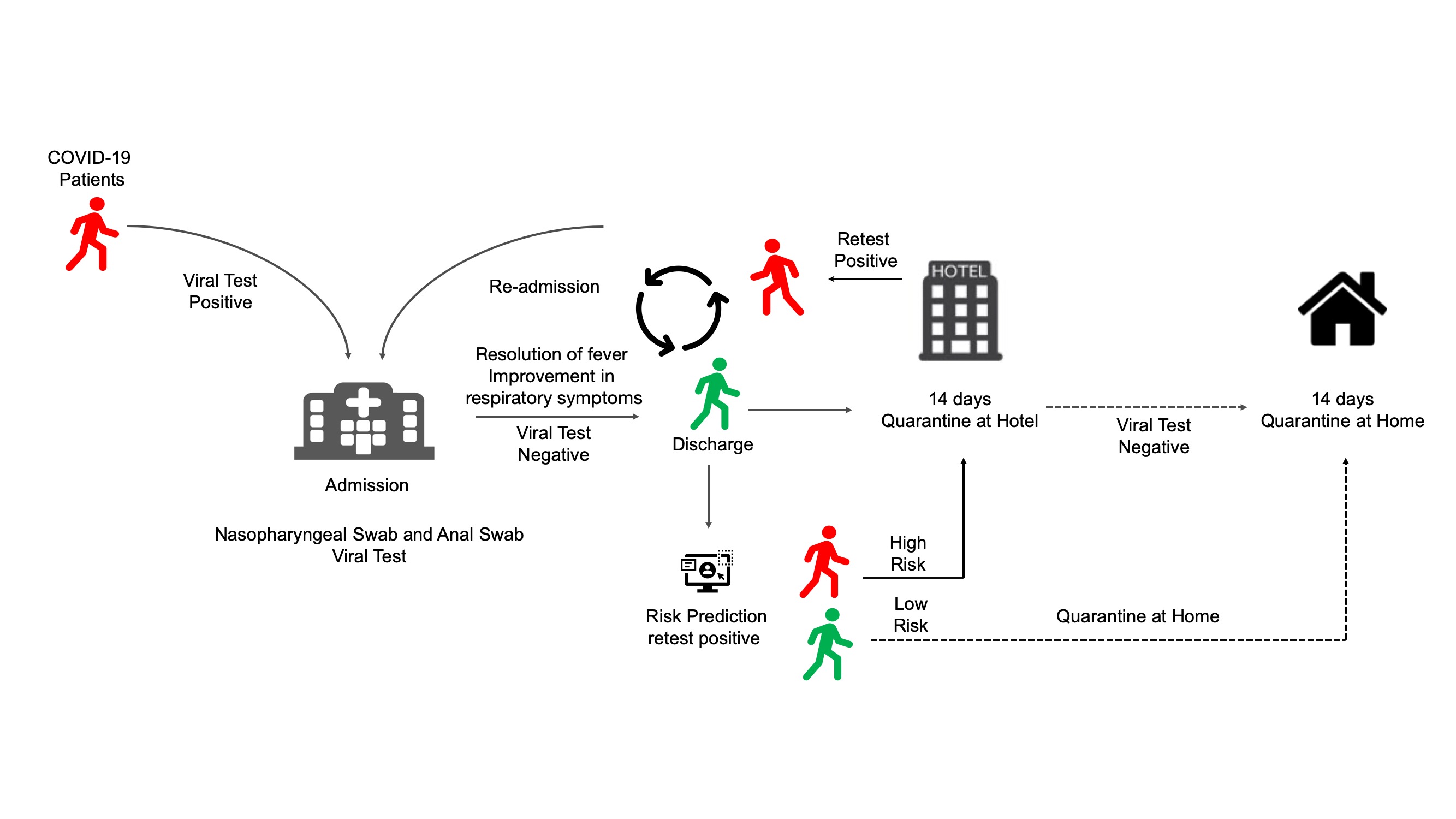


**Figure S2.**

**A**


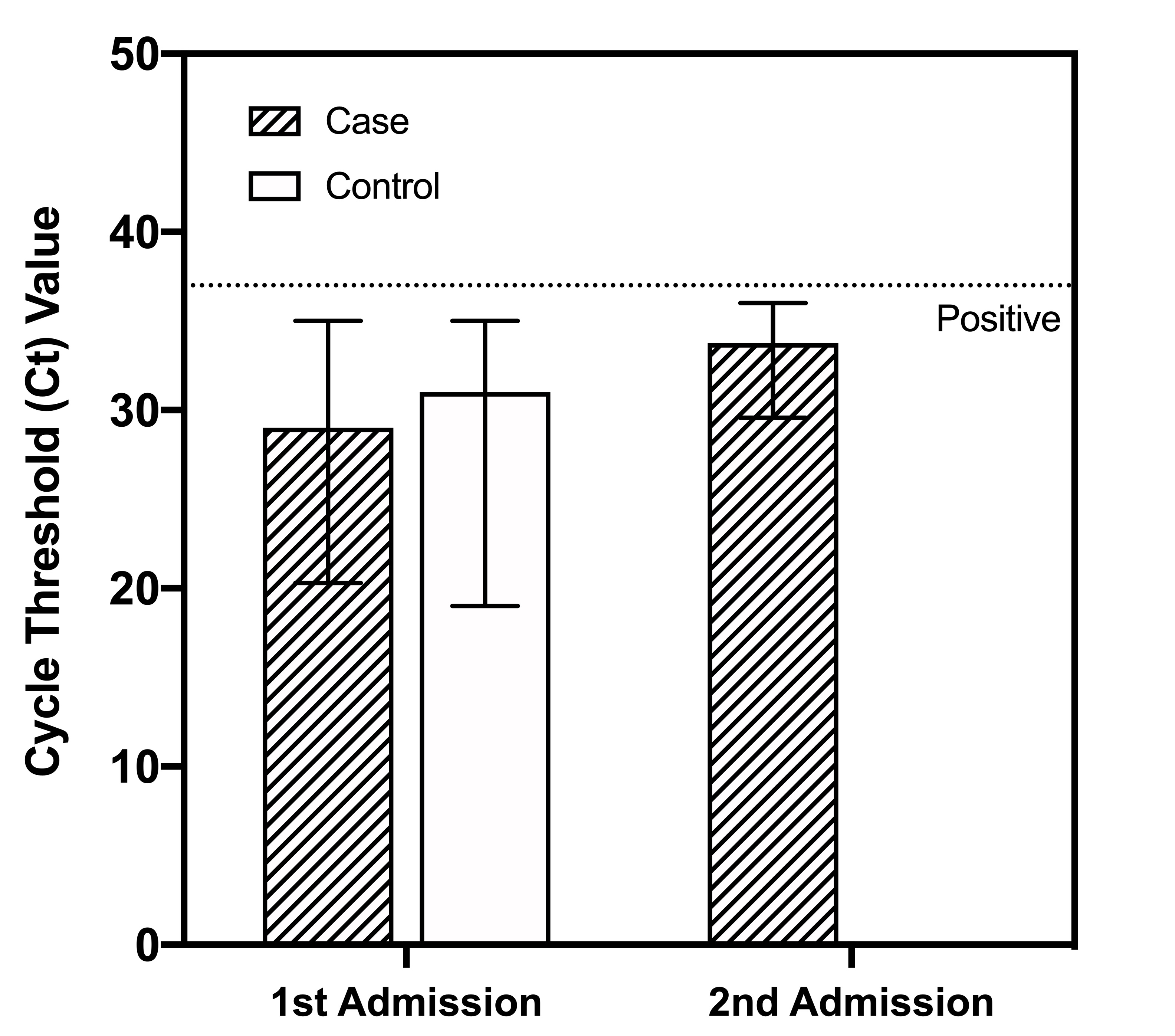


**B**

**
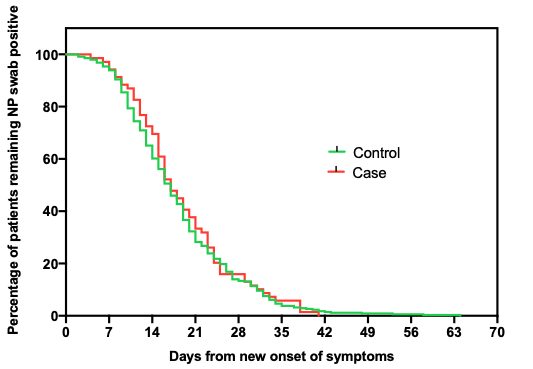
**

**Figure S3**.


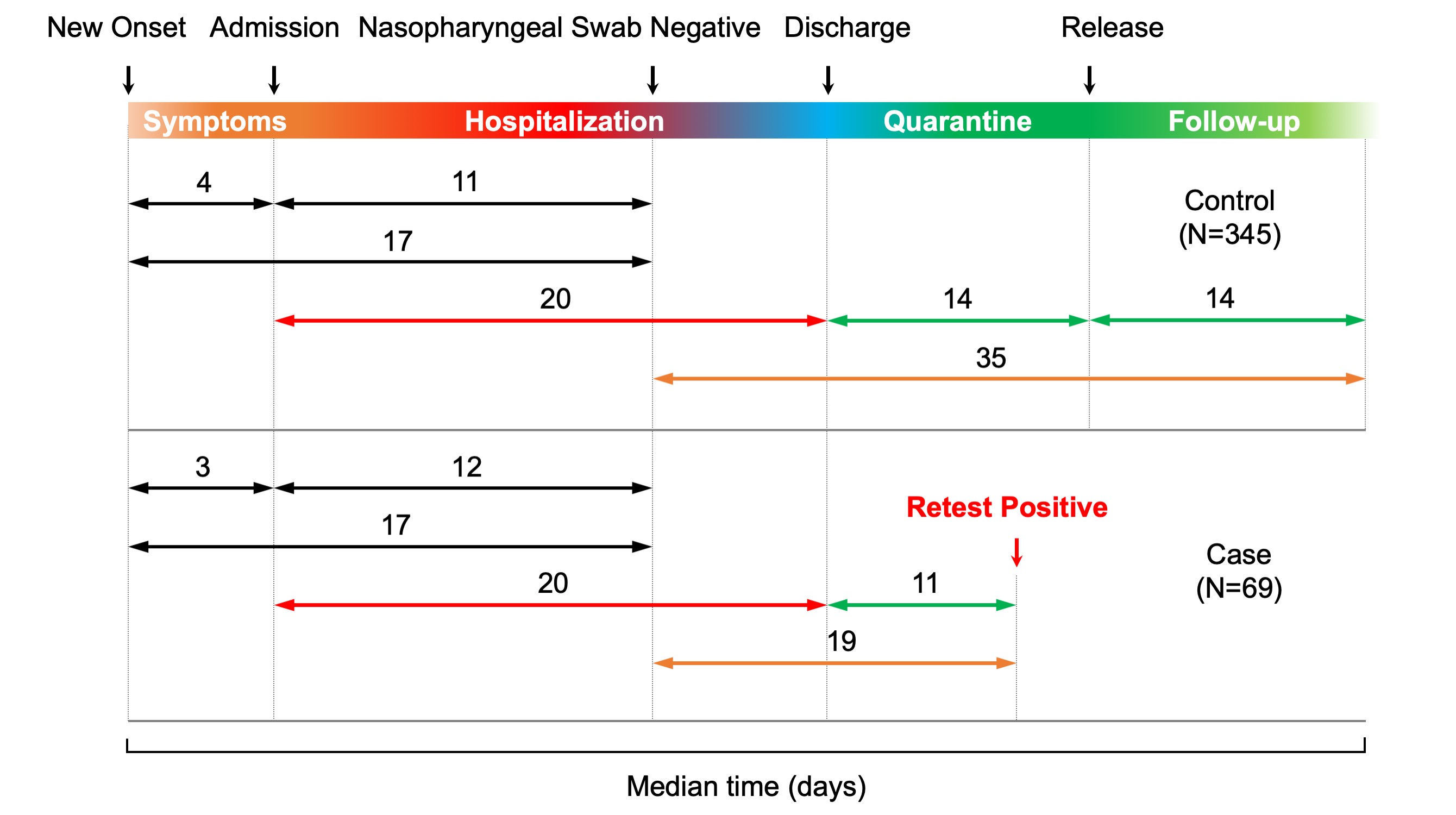


**Figure S4**.


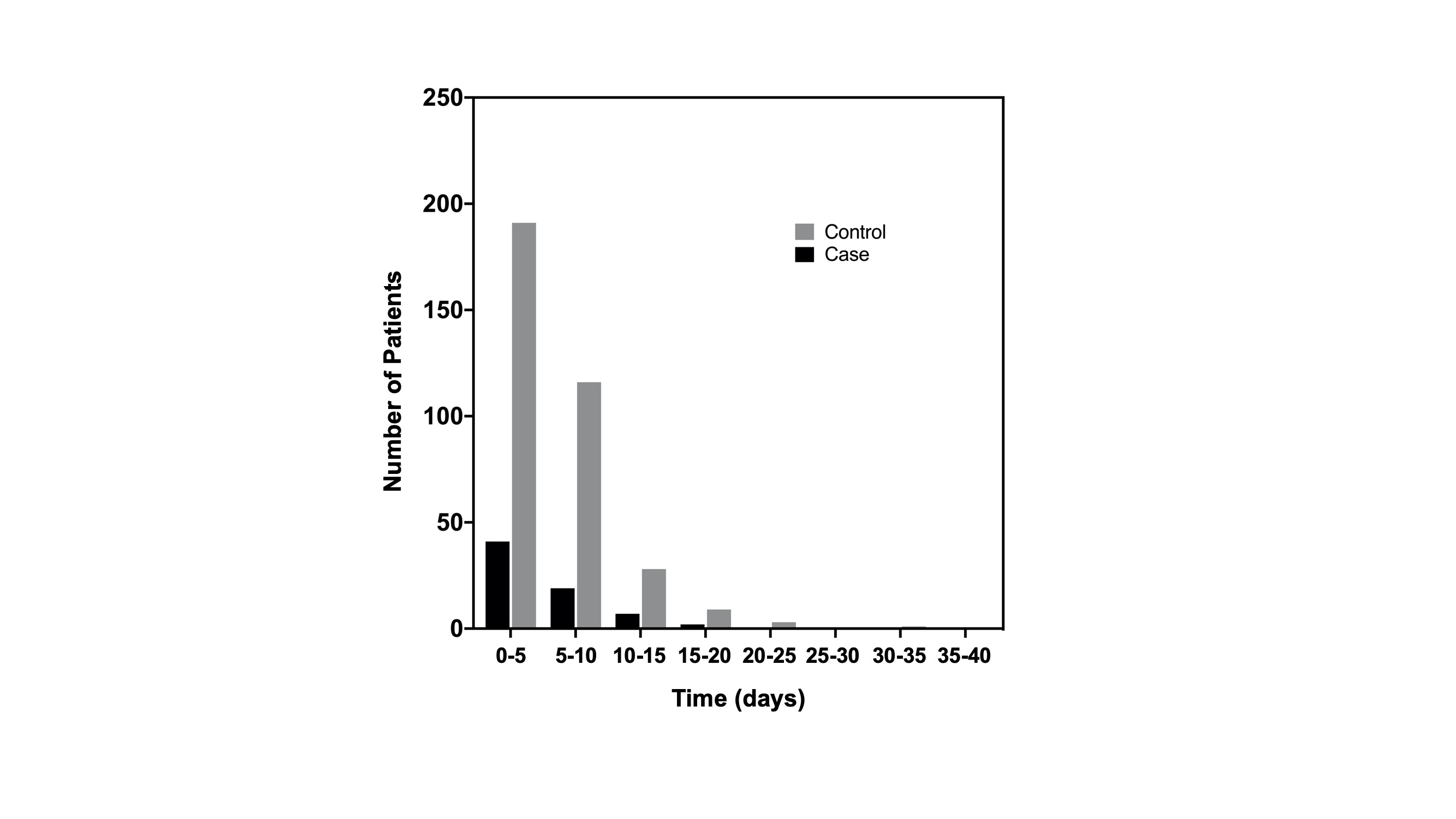


**Figure S5**.


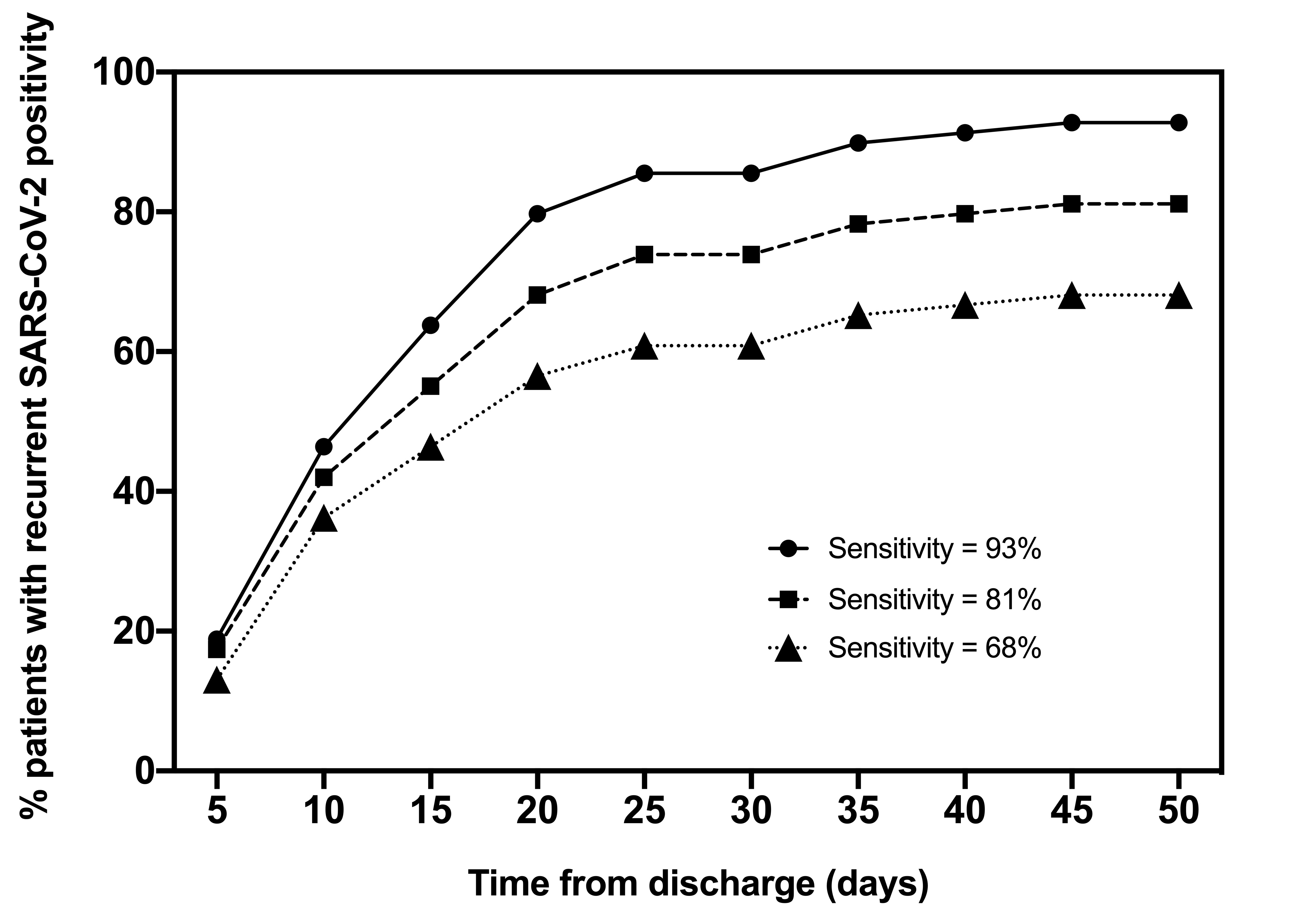


**Figure S6**.


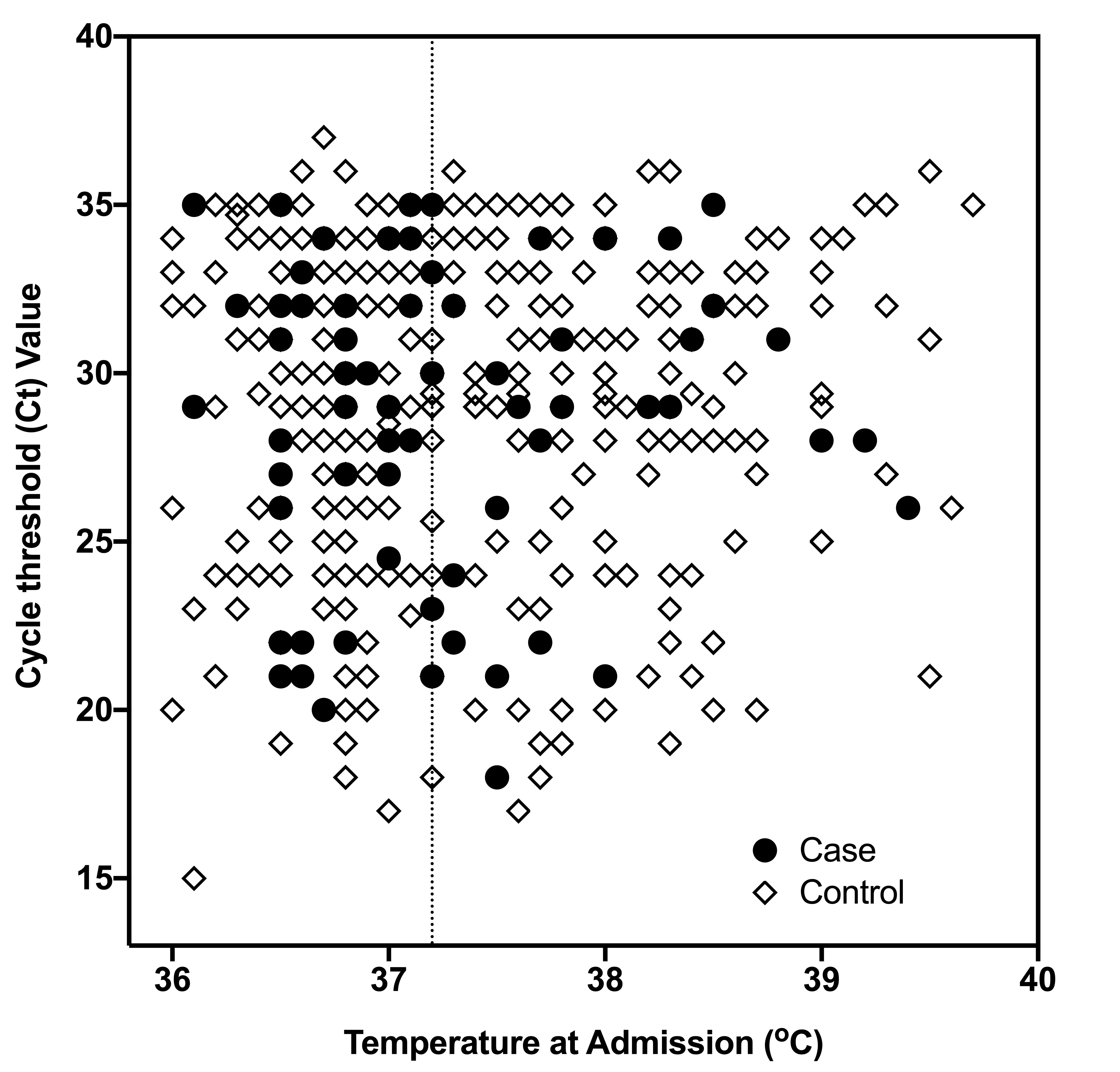


**Table S1**.

| **Feature** | | **Importance measure** | **Odds Ratio (95% CI)** | ***P* Value** |
| --- | --- | --- | --- | --- |
| Demographics | Age, yr | 0.083 (0.049, 0.128) | 0.615 (0.462, 0.812) | 0.0007 |
|  | BMI, kg/m^2^ | 0.055 (0.025, 0.087) | 0.535 (0.206, 1.374) | 0.1956 |
| First test results during hospitalization | White blood cell, × 10^9^/L | 0.052 (0.023, 0.110) | 1.227 (0.950, 1.575) | 0.1064 |
|  | Lymphocyte, × 10^9^/L | 0.041 (0.017, 0.113) | 1.210 (0.944, 1.544) | 0.1222 |
|  | Procalcitonin, ng/mL | 0.048 (0.026, 0.068) | 56.757 (2.2, 2179.386) | 0.0207 |
|  | Partial pressure of oxygen, mmHg | 0.055 (0.024, 0.089) | 1.325 (0.944, 1.869) | 0.1021 |
|  | Ct value of NP swab RT-PCR | 0.036 (0.005, 0.055) | 0.781 (0.602, 1.016) | 0.0625 |
| Lowest test results during hospitalization | Cholinesterase, U/L | 0.118 (0.052, 0.152) | 1.861 (1.349, 2.604) | 0.0002 |
|  | Fibrinogen, g/L | 0.075 (0.019, 0.097) | 0.747 (0.553, 0.994) | 0.0503 |
|  | Albumin, g/L | 0.064 (0.045, 0.090) | 1.340 (0.935, 1.948) | 0.1174 |
|  | Prealbumin, ng/L | 0.054 (0.033, 0.070) | 1.585 (1.189, 2.122) | 0.0017 |
|  | Calcium, mmol/L | 0.045 (0.017, 0.087) | 1.301 (1.042, 1.607) | 0.0163 |
|  | Creatinine, μmol/L | 0.025 (0.016, 0.074) | 0.824 (0.612, 1.103) | 0.1971 |
|  | EGFR, ml/min/1.73m^2^ | 0.054 (0.019, 0.080) | 1.327 (1.020, 1.719) | 0.0322 |
|  | Ct value of NP swab RT-PCR | 0.033 (0.009, 0.085) | 0.712 (0.538, 0.943) | 0.0172 |
| Highest test results during hospitalization | Total bilirubin, μmol/L | 0.089 (0.077, 0.178) | 0.686 (0.456, 0.987) | 0.0551 |
|  | Lactate dehydrogenase, U/L | 0.054 (0.034, 0.112) | 0.707 (0.418, 1.150) | 0.1782 |
|  | Alkaline phosphatase, U/L | 0.020 (0.009, 0.048) | 1.168 (0.913, 1.473) | 0.1908 |

**Table S2**.

| **Threshold of high-risk** | **Sensitivity** | **Specificity** |
| --- | --- | --- |
| Threshold 1 | 100% | 12.8% |
| Threshold 2 | 93% | 36.8% |
| Threshold 3 | 81% | 59.7% |
| Threshold 4 | 68% | 70.4% |
| Threshold 5 | 62% | 79.1% |

**Text S1.**

The leading features in the algorithm predicting recurrence of polymerase chain reaction (PCR) positivity included: age; body mass index (BMI); lowest levels of the blood laboratory tests during hospitalization for cholinesterase, fibrinogen, albumin, prealbumin, calcium, estimated glomerular filtration rate (eGFR), creatinine; highest levels of the blood laboratory tests during hospitalization for total bilirubin, lactate dehydrogenase, and alkaline phosphatase; the first test results during hospitalization for partial pressure of oxygen, total white blood cell and lymphocyte counts, blood procalcitonin; and the first test result and lowest level of cycle threshold (Ct) value of the nasopharyngeal (NP) swab reverse transcription (RT) PCR results.

The model identified serum concentrations of cholinesterase, calcium, and eGFR as predictors, and the elevation of the three markers were associated the increasing risk of recurrence of PCR positivity. Previous studies found that cholinesterase, calcium, and eGFR were all associated with the severity of COVID-19. A study in Zunyi, China found that cholinesterase levels in mild coronavirus disease 2019 (COVID-19) cases were significantly higher than those in severe cases ^1^. Shanghai Public Health Clinical Centre found that among 198 COVID-19 patients, non-ICU patients had significantly higher levels of calcium than those who were admitted to ICU ^2^.A study on 701 patients in Tongji Hospital, China revealed that kidney disease was associated with in-hospital deaths of patients with COVID-19. Furthermore, eGFR, a marker of kidney dysfunction, was found significantly lower in severe patients with COVID-19^3^.All these findings suggested that increased levels of cholinesterase, calcium, and eGFR were associated with mild COVID-19. In our study, mild or moderate patients had more chance to get recurrence of PCR positivity. The associations of the blood markers and the recurrence of PCR positivity outcomes revealed by our model therefore were consistent with results from other centers.

1. Xiang J. et al. Potential biochemical markers to identify severe cases among COVID-19 patients. Preprint at <https://www.medrxiv.org/content/10.1101/2020.03.19.20034447v1> (2020).
2. Cao M. et al. Clinical Features of Patients Infected with the 2019 Novel Coronavirus (COVID-19) in Shanghai, China. Preprint at <https://www.medrxiv.org/content/10.1101/2020.03.04.20030395v1> (2020).
3. Cheng Y. et al. Kidney disease is associated with in-hospital death of patients with COVID-19. *Kidney International*. **97**, 829-38. <https://doi.org/10.1016/j.kint.2020.03.005> (2020).
